# Natural pollen exposure increases in a dose‐dependent way Fraction of exhaled Nitric Oxide (FeNO) levels in patients sensitized to one or more pollen species

**DOI:** 10.1002/clt2.12096

**Published:** 2022-02-06

**Authors:** Mario Olivieri, Pierpaolo Marchetti, Nicola Murgia, Morena Nicolis, Lorena Torroni, Gianluca Spiteri, Marcello Ferrari, Alessandro Marcon, Giuseppe Verlato

**Affiliations:** ^1^ Unit of Occupational Medicine, Department of Diagnostics and Public Health Policlinico “G. Rossi” Verona Italy; ^2^ Unit of Epidemiology and Medical Statistics, Department of Diagnostics and Public Health University of Verona Verona Italy; ^3^ Section of Occupational Medicine, Respiratory Diseases and Toxicology University of Perugia Perugia Italy; ^4^ Unit of Hygiene and Preventive, Environmental and Occupational Medicine, Department of Diagnostics and Public Health University of Verona Verona Italy; ^5^ Department of Medicine, Section of Respiratory Diseases University of Verona Verona Italy

**Keywords:** epidemiology, Fractional exhaled Nitric Oxide, natural pollen exposure, pollen‐related allergic symptoms, polysensitization

## Abstract

**Background:**

Co‐exposures and polysensitization to several pollen species are very common in real life practice. However, little information exists on allergic symptoms and airway inflammation related to natural pollen exposure in large general population samples.

**Objective:**

To assess the combined effect of sensitization and/or exposure to one or more pollen species on Fraction of exhaled Nitric Oxide (FeNO) levels.

**Methods:**

Within Gene Environment Interactions in Respiratory Diseases (GEIRD) multicase‐control study, 1070 adults from the general population of Verona, Italy, underwent a clinical evaluation including standardized interview, spirometry, skin prick test to inhalants and FeNO measurement. Pollen exposure was assumed, when the mean pollen concentration in the previous week was above the cutoff established by the Italian Aerobiological Monitoring Network.

**Results:**

Subjects sensitized to one or more pollen species were respectively 15.5% and 29.6%. FeNO levels were directly related to the number of both pollen species around and pollen‐related sensitizations. Median FeNO levels were directly related to number of pollen species around and pollen sensitization. FeNO levels increased from 15.4 ppb (p. 25–p. 75 = 9.9–21.0) outside the pollen season to 17.5 ppb (11.2–30.5) when there were ≥3 pollen species around. Likewise FeNO levels rose from 14.8 ppb (10.0–22.3) in not sensitized subjects, to 16.7 (10.1–25.0) in monosensitized and further to 20.4 (12.3–40.6) in poly‐sensitized. According to multivariable quantile regression, median FeNO was 17.9 ppb higher (p. 25–p. 75 = 12.5–23.3) for subjects sensitized and exposed to more than one pollen species, compared to subjects who were neither sensitized nor exposed. Differences in FEV1/FVC between groups were less pronounced (*−*2.0%, −4.1 to 0.1). Median FeNO level was 15.1 ppb (p. 25–p. 75 = 10.0–23.2) in subjects without pollen‐related symptoms, 17.8 ppb (12.1–40.2) in those with nasal symptoms only, and 22.7 ppb (14.7–43.0) in those with asthma‐like symptoms (*p* < 0.001).

**Conclusion and clinical relevance:**

Airways inflammation, evaluated by FeNO, increases in dose‐dependent manner from subjects monosensitized to pollen species to those poly‐sensitized, especially when asthma‐like symptoms on pollen exposure are also reported. This should be considered by allergists during natural pollen seasons when evaluating both pulmonary function and airways inflammation.

## 1 INTRODUCTION

Over the last decades, population‐based studies showed an increased prevalence of atopic diseases in developed countries, particularly in children and young adults.^1^, ^2^, ^3^ Several longitudinal studies in birth cohorts reported that allergic sensitization increases with age from childhood to adulthood.^4^, ^5^ In this context, polysensitization, that is, sensitization to an increasing number of allergens in the same patient, has become a common feature, and the percentage of polysensitization among allergic patients ranges from 20% to 90%.^6^, ^7^, ^8^ Polysensitization is an immunological phenomenon that is clinically significant and relevant from an epidemiological point of view, although to be sensitized does not mean to be allergic when exposed to the related allergen. Furthermore, pollen co‐exposure is also becoming more prolonged and more intense, as climatic changes are anticipating pollen season^9^ and increasing pollen production and allergenicity.^10^ Co‐exposure to several allergens in poly‐sensitized patients is associated with more severe nasal and asthma‐like symptoms, with respect to mono‐sensitized ones.^11^

An easy‐to‐assess, reproducible biomarker, particularly useful in evaluating patients with type‐2 airway inflammation, is Fraction of exhaled Nitric Oxide (FeNO), that is, usually increased in IgE‐mediated allergic inflammation. A persistent late increase in FeNO levels has been reported after an acute specific inhalation challenge^12^ and also after repeated nasal allergen provocation outside the pollen season.^13^ Accordingly, children with allergic asthma sensitized to grass pollen present a significant increase in individual FeNO levels during the natural pollen season with respect to preseason baseline values.^14^

The aim of the present study was to evaluate the impact of sensitization and exposure to one or more pollen species on FeNO levels. This topic was investigated in a population‐based sample, taking into account airborne pollen species levels in the week before the clinical evaluation.

## 2 MATERIAL AND METHODS

### 2.1 Study design

Gene Environment Interactions in Respiratory Diseases (GEIRD) is a multicase‐control study on respiratory health, in which cases and controls were identified through a two‐stage screening process in pre‐existing cohorts and in new random samples from the Italian general population.^15^

In the first stage (2007–2010), eligible subjects were administered a screening questionnaire on respiratory symptoms. In the second stage (2008–2016) all participants with symptoms suggestive of asthma, chronic obstructive pulmonary disease (COPD) or chronic bronchitis (CB) and a random sample of subjects without respiratory symptoms or with symptoms suggestive of rhinitis, were referred to clinical centres to undergo the ‘phenotypization’ protocol. Questionnaires and descriptive characteristics of the GEIRD study are available on the web site http://www.geird.org, while protocols are available on the http://biometria.univr.it/sesm/progetti.html.

The present study was based on clinical data from participants aged 20‐64 years from the centre of Verona. Out of 5056 subjects who filled in the screening questionnaire, 2961 were invited to the clinical examination, and 1322 of them were seen in clinic (response rate = 45%). The present study was performed on 1070 subjects, who had information on FeNO and/or lung volumes.

### 2.2 Data collection

Clinical data were obtained by means of a structured medical interview through the Clinical Questionnaire, a modification of the European Community Respiratory Health Survey (ECRHS) questionnaire (http://www.ecrhs.org) including detailed questions on respiratory symptoms, medical history, use of medication, health services, occupation, socio‐economic status, home environment and lifestyle. With regard to the smoking habits, subjects were classified as follows: 1) current smokers, if they reported to have smoked at least 20 packs of cigarettes or 12 oz (360 g) of tobacco in a lifetime, or at least one cigarette per day or one cigar per week for 1 year, and also during the last month; 2) ex‐smokers, if they had smoked at least one cigarette per day or one cigar per week for 1 year, but not in the last month; 3) never smokers, otherwise^15^, ^16^

### 2.3 Definition of cases and controls

Based on reported symptoms in the first stage and results of the clinical tests, subjects were classified into cases of asthma (irrespective of rhinitis), cases of rhinitis alone, and controls, according to the GEIRD protocol.^17^ Subjects with CB/COPD (*n* = 134) were excluded from the present study, as well as subjects who did not have enough information to properly define case/control status (*n* = 118). Hence the analyses were performed on 1070 subjects, classified as follows:

‐ cases of asthma, irrespective of rhinitis (*n* = 315): (1) self‐reported asthma, plus one among having had an asthma attack in the last 12 months or current use of medications for asthma; or (2) asthma‐like symptoms or use of asthma medicines in the last 12 months, plus one among positive methacholine challenge test (PD20 < 1 mg) or pre‐bronchodilator forced expiratory volume 1 s (FEV_1_)/forced vital capacity (FVC) < 70% or < lower limit of normal (LLN) with a positive reversibility test (i.e., FEV_1_ > 12% and >200 ml);
‐ cases of rhinitis alone (*n* = 312): subjects without asthma, with lifetime nasal allergies, including ‘hay fever’; lifetime problem with sneezing, or a runny or a blocked nose (without cold/flu); recurrent nasal/eye symptoms in the presence of dust, pollens or animals.
‐ controls (*n* = 443): subjects without asthma or rhinitis, who did not report other respiratory symptoms or diseases, and had both pre‐bronchodilator FEV_1_/FVC ≥70% and ≥LLN and FEV_1_ >70% predicted.

### 2.4 Pulmonary function tests and FeNO levels

Airway inflammation was assessed by measuring FeNO (ppb at 50 ml/sec) using a chemiluminescence analyser (CLD88, Ecomedics), according to international guidelines, and hence before spirometry.^18^, ^19^ The influence of ambient NO levels was excluded by placing a NO‐scrubbing filter in the inspiratory limb of the collection apparatus.

Pulmonary function was measured with Biomedin spirometer as the ratio of forced expiratory volume in 1 s to FVC (FEV1/FVC). Forced expiratory volume in 1 s (FEV1) and FVC were measured according to the American Thoracic Society reproducibility criteria.^20^ In smokers, the measurement of FeNO and spirometry was performed at least 1 h after smoking the last cigarette.

### 2.5 Total serum IgE

Total serum IgE level was measured using the Pharmacia CAP system (Uppsala, Sweden).

### 2.6 Symptoms related to seasonal allergens exposure

Asthma‐like symptoms related to pollen exposure were defined by a positive answer to at least one of the following questions: ‘When you are near trees, grass or flowers, or when there is a lot of pollen about, do you ever start to wheeze?’, ‘get a feeling of tightness in your chest?’, ‘start to feel short of breath?’. A subject was deemed to suffer from nasal symptoms related to pollen exposure if he/she answered ‘yes’ to the question ‘get a runny or stuffy nose or start to sneeze?’.

### 2.7 Skin prick tests and exposure to allergens

Skin prick tests were performed with a panel of inhaled allergens (*Cupressus arizonica*, *Graminacee mix*, *Artemisia vulgaris*, *Ambrosia artemisifolia*, *Alternaria tenuis*, *Parietaria Judaica*, *Corylus avellana*, *Olea europea*, *Betula verrucosa*, *Cladosporium herbarum*, *Dog dander*, *Cat hair*, *Dermatophagoides pteronyssinus*, *Dermatophagoides farinae*) (ALK diagnostics, Denmark). Wheal diameter of ≥3 mm was considered as positive reaction.^21^

Data on the daily air concentrations of seven pollens from four vegetal families (*Poaceae*, *Urticaceae*, *Oleaceae*, *Cupressaceae*), one subfamily (*Coryloideae*), and two genera (*Betula* and *Ambrosia*) were collected from local monitoring stations for the years when the clinical examinations were conducted.^22^ For pollen taxa, we calculated the mean pollen concentration during the week before a subjects' clinical examination. A subject was considered exposed to a specific pollen if the mean pollen concentration was above the cutoff established by the Italian Aerobiological Monitoring Network (Rete Italiana di Monitoraggio in Aerobiologia) of the Italian Association of Aerobiology (Associazione Italiana di Aerobiologia) (http://www.ilpolline.it/). In details, the thresholds in grains/m^3^ were: *Poaceae*, >10; *Urticaceae*, >20; *Cupressaceae*, >30; *Corylaceae* and *Betula*, >16; and *Ambrosia and Oleaceae*, >5.^22^ Subjects were considered polyexposed when more than one pollen species presented a concentration above the reported cutoffs.

Considering exposure and sensitization to pollen species, subjects were classified as follows: 1) neither exposed nor sensitized to any seasonal pollen species (E−/S−), 2) Not exposed but sensitized (E−/S+) or exposed but not sensitized (E+/S−) to any seasonal pollen species, 3) Exposed and sensitized to one pollen species (E+/S+), 4) Both exposed and sensitized to more than one pollen species (E++/S++).

### 2.8 Statistical analysis

Descriptive statistics was accomplished using percentage for categorical variables and median with I and III quartiles for quantitative variables. Wilcoxon signed‐rank test and Kruskal‐Wallis test were used to test differences across groups where appropriate. Post hoc analysis was accomplished adjusting for multiple testing bias.

The combined influence of exposure and sensitization to pollen species on FeNO level, FEV_1_/FVC and total IgE levels was assessed by multivariable quantile regression, controlling for sex, age, Body Mass Index (BMI) (coded as underweight, normal weight, overweight and obese), age at completed education (<16 years, ≥16 years), smoking status (never smokers, former smokers, current smokers) and case‐control status (controls, rhinitis only, asthma with/without rhinitis). The model was also adjusted for sensitization to animal dander and dust mites coded as not sensitized/sensitized to at least one allergen.

A sensitivity analysis was also performed by coding FeNO levels as normal (<25 ppb), intermediate (25–50 ppb) and elevated (>50 ppb) according to Dweik et al.^18^

The analysis was done using STATA 15.0 (StataCorp, Texas, USA) and statistical significance was set at *p*‐value <0.05.

## 3 RESULTS

The 1070 subjects participating in the study had a median age of 44.6 years (Q1–Q3: 37.5–51.8) and a slight majority of women (Table 1). The proportion of mono‐sensitized (13.0%) and polysensitized (38.2%) subjects by case‐control status is reported in Table 2. Proportions of subjects sensitized to one or more seasonal pollen species were respectively 15.5% and 29.6% (Table 2), 30% of controls were sensitized to airborne allergens tested, and the proportion of mono‐ and poly‐sensitized were about the same. Sixty percent of rhinitis cases were sensitized, mostly poly‐sensitized. Two thirds of asthmatic cases were poly‐sensitized to whatever allergen, while only a minority (<10%) were monosensitized.

**TABLE 1:** Median (p. 25–p. 75) of Fraction of exhaled Nitric Oxide (FeNO), Forced expiratory volume in 1 s (FEV1)/forced vital capacity (FVC) and total IgE levels as a function of demographic and lifestyle characteristics

Variables	*n* (%)	FeNO (ppb) *n* = 856		FEV1/FVC *n* = 1067		Total IgE (kU/L) *n* = 787	
		Median^a^ (p. 25–p. 75)	p*	Median^a^ (p. 25–p. 75)	p*	Median^a^ (p. 25–p. 75)	p*
Sex			**<0.001**		**0.041**		**<0.001**
Women	557 (52.1)	14.7 (9.5–21.0)		82.5 (78.5–86.8)		32.3 (13.5–96.9)	
Men	513 (47.9)	18.6 (11.8–32.8)		81.3 (76.5–85.3)		60.7 (21.7–152.5)	
Smoking status^b^			**<0.001**		**0.020**		**0.011**
Never smokers	548 (51.3)	17.0 (11.3–26.7)		82.9 (78.3–87.3)		40.0 (15.7–119)	
Past smokers	303 (28.3)	17.7 (11.1–31.3)		81.2 (77.4–85.0)		36.5 (14.8–115)	
Current smokers	218 (20.4)	12.9 (8.5–17.4)		80.8 (77.3–85.0)		67.0 (22.7–156)	
Age at completed education^b^			0.886		**0.001**		0.702
<16 years	147 (13.8)	16.0 (10–23.5)		80.5 (76.6–85.4)		42.8 (16.8–121)	
≥16 years	921 (86.2)	16.3 (10.6–26.9)		82.2 (77.9–86.3)		36.5 (14.8–123.5)	
Age (years)			0.674		**<0.001**		0.211
20–44	548 (51.2)	16.1 (10.6–26.5)		83.4 (79.1–87.8)		48.2 (17.5–126)	
45–64	522 (48.8)	16.3 (10.3–25.1)		80.6 (76.5–84.9)		37.5 (16.2–116)	
BMI (kg/m^2^)^b^			**0.008**		0.910		**0.033**
<18.5	21 (2.0)	17.1 (12.7–36.2)		84.3 (78.2–92.1)		36.5 (9.6–82.7)	
18.5–24.9	587 (55.1)	15.0 (9.9–23.5)		82.3 (77.7–86.6)		40.3 (15.1–110)	
25.0–29.9	333 (31.2)	18.3 (11.5–29.9)		81.1 (77.0–84.9)		46.3 (17.9–126)	
≥30	125 (11.7)	17.2 (11.2–28)		83.2 (79.4–87.1)		65.7 (22.1–176.5)	
Case‐control status			**<0.001**		**<0.001**		**<0.001**
Controls	443 (41.4)	15.4 (10.4–22.6)		83.7 (79.7–87.7)		26.8 (12.5–77.2)	
Rhinitis only	312 (29.2)	14.6 (9.5–24.5)		82.2 (78.0–86.3)		47.9 (18.5–110)	
Asthma with/without rhinitis	315 (29.4)	19.9 (12.4–40.7)		79.5 (75.6–83.3)		94.6 (33.9–240)	

*Note*: Bold signifies sex vs. FEV1/FVC *p*‐value “0.041”.

^a^ Crude values.

^b^ Information on smoking status, age at complete education and Body Mass Index (BMI) was missing in 1, 2, and 4 subjects respectively.

*p‐values were computed by Kruskal‐Wallis test for case‐control status, and by quantile regression, adjusting for case‐control status, for all other explanatory variables.

**TABLE 2:** Proportion of not sensitized, mono‐sensitized and poly‐sensitized among controls, cases of rhinitis only, cases of asthma with or without rhinitis. Sensitization was evaluated by considering first only seasonal allergens, and then perennial allergens and all allergens tested

Seasonal allergen	Controls		Cases rhinitis only		Cases of asthma/rhinitis		Overall	
	*N*	%	*n*	%	*N*	%	N	%
Seasonal pollen species								
Not sensitized	339	76.5	145	46.5	103	32.7	587	54.9
Monosensitized	67	15.1	62	19.9	37	11.8	166	15.5
Polysensitized	37	8.4	105	33.6	175	55.5	317	29.6
Perennial allergens^a^								
No sensitized	382	86.4	229	73.9	147	47.1	758	71.2
Monosensitized	51	11.5	52	16.8	86	27.6	189	17.8
Polysensitized	9	2.1	29	9.3	79	25.3	117	11.0
All allergens tested								
No sensitized	313	70.7	128	41.0	81	25.7	522	48.8
Monosensitized	60	13.5	51	16.4	28	8.9	139	13.0
Polysensitized	70	15.8	133	42.6	206	65.4	409	38.2

^a^ Information on perennial allergens was missing in one subject.

FeNO and total IgE levels were significantly higher in men than women, in subjects with asthma than in those with rhinitis only or controls. Current smokers had lower FeNO and higher total IgE levels than either past or never smokers. FeNO levels were lower in normoweight subjects than underweight or overweight/obese people (Table 1).

FEV_1_/FVC was lower in men than women, in people with low than high education, in asthmatics than in subjects with rhinitis only or controls. FEV_1_/FVC was lower at older ages and for past and current smokers, compared to never smokers, and it was the lowest in overweight people (Table 1).

FeNO was significantly higher during the natural pollen season, when two or more allergenic pollen species were simultaneously present, while IgE levels were not significantly affected by exposure to pollens. FeNO and IgE levels significantly increased from people not sensitized to pollen to monosensitized and further to multisensitized subjects. FEV_1_/FVC progressively decreased with increasing number of pollen species around and with sensitization to two or more pollen species (Table 3).

**TABLE 3:** Median (p. 25–p. 75) Fraction of exhaled Nitric Oxide (FeNO), Forced expiratory volume in 1 s (FEV1)/forced vital capacity (FVC) and total IgE levels as a function of exposure to pollen species at the time of clinical assessment, and pollen‐related sensitization

Variables		FeNO (ppb) *n* = 856		FEV1/FVC *n* = 1067		Total IgE (kU/L) *n* = 787	
	*n* (%)	Median^a^ (p. 25–p. 75)	p*	Median^a^ (p. 25–p. 75)	p*	Median^a^ (p. 25–p. 75)	p*
Exposure to pollen species			**0.023**		**0.013**		0.157
No pollen around	316 (29.5)	15.4 (9.9–21.0)		82.8 (78.9–86.4)		42.3 (17.7–126.0)	
1 pollen species	261 (24.4)	15.1 (10.1–27.3)		82.7 (77.8–88.0)		51.5 (19.9–115.0)	
2 pollen species	161 (15.1)	16.0 (10.9–27.5)		81.7 (76.5–85.3)		40.2 (15.1–165.0)	
≥3 pollen species	332 (31.0)	17.5 (11.2–30.5)		81.2 (77.5–85.1)		37.1 (15.1–119.0)	
Pollen‐related sensitization			**<0.001**		0.711		**<0.001**
No	587 (54.9)	14.8 (10.0–22.3)		82.6 (78.1–86.8)		25.0 (11.6–62.7)	
Mono‐sensitization	166 (15.5)	16.7 (10.1–25.0)		82.3 (78.6–86.3)		51.98 (20.0–136.5)	
Multi‐sensitization	317 (29.6)	20.4 (12.3–40.6)		80.5 (76.1–84.7)		123.0 (57.5–284.5)	

*Note*: Bold signifies exposure to pollen species vs. FeNO *p*‐value “0.023” and vs. FEV1/FVC *p*‐value “0.013”.

^a^ Crude values.

*p‐values were computed by quantile regression, adjusting for case‐control status.

**FIGURE 1:**
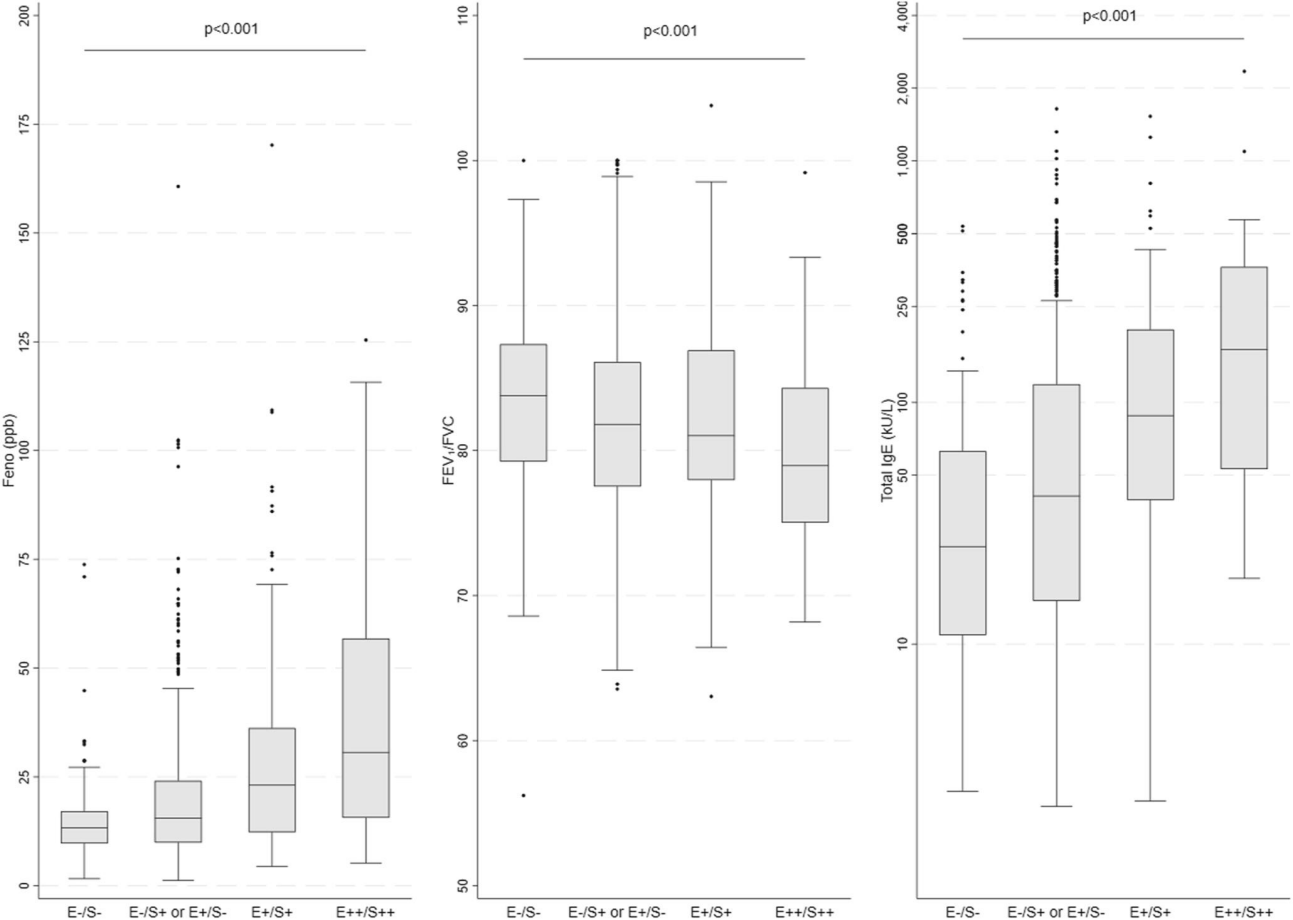
Box and whisker plot of the Fraction of exhaled Nitric Oxide (FeNO) levels, Forced expiratory volume in 1 s (FEV1)/forced vital capacity (FVC) and total IgE levels stratified by combined sensitization and exposure to seasonal allergens at the time of clinical assessment. E−/S− = not exposed/not sensitized; E−/S+ or E+/S− = not exposed/sensitized or exposed/not sensitized; E+/S+ = Exposed and sensitized to one pollen species; E++/S++ = Exposed and sensitized to more than one pollen species. *p*‐values were computed by Kruskal‐Wallis test

The distribution of FeNO and total IgE levels was skewed to the right. FeNO progressively increased from controls (people not exposed and not sensitized) to people either exposed but not sensitized (E+/S−) or sensitized but not exposed (E−/S+). Median FeNO nearly doubled in people exposed and sensitized to one pollen species (E+/S+) and nearly tripled in those exposed and sensitized to two or more pollen species (E++/S++). Total IgE levels presented a similar pattern, as median value (p. 25–p. 75) were 25 kU/l (11–63) in controls, increased to 41 kU/l (15–119) in people either sensitized or exposed, and to 88 kU/l (40–200) in subjects' mono‐sensitized and mono‐exposed, and 166 kU/L (53–364) in those poly‐sensitized and poly‐exposed. FEV1/FVC progressively decreased from controls to people either exposed or sensitized, and further to people exposed and sensitized to one pollen species, and further to those exposed and sensitized to two or more pollen species (Figure 1).

In multivariable analysis sensitization to pollen species did not significantly increase FeNO levels in the absence of related exposure (Table 4). Conversely the same occurred in people exposed but not sensitized to pollen species. As expected, pollen exposure was associated with a significant increase in FeNO levels in sensitized subjects, which became more pronounced when two or more pollen species were simultaneously involved. This pattern did not change when adjusting for sensitization to perennial allergens. A similar trend was recorded as regards total IgE levels. The effect of sensitization/related exposure to pollen species on FEV_1_/FVC was less manifest, becoming clearly evident in subjects simultaneously sensitized and exposed to two or more pollen species.

**TABLE 4:** Impact of combined exposure/sensitization to seasonal allergens on median Fraction of exhaled Nitric Oxide (FeNO), Forced expiratory volume in 1 s (FEV1)/forced vital capacity (FVC) and total IgE levels

Combined sensitization and exposure to pollen species	FeNO (ppb)		FEV_1_/FVC (%)		Total IgE (kU/L)	
	Coefficient^a^ (95% CI)	Coefficient^b^ (95% CI)	Coefficient^a^ (95% CI)	Coefficient^b^ (95% CI)	Coefficient^a^ (95% CI)	Coefficient^b^ (95% CI)
Not sensitized/not exposed	0 (ref.)	0 (ref.)	0 (ref.)	0 (ref.)	0 (ref.)	0 (ref.)
Sensitized/not exposed or not sensitized/exposed	2.3 (−0.7; 5.2)	2.4 (−0.6; 5.3)	−1.6 (−2.8; −0.4)**	−1.5 (−2.7; −0.3)*	4 (−14; 23)	2 (−17; 22)
Sensitized and exposed to one pollen species	7.6 (3.5; 11.6)***	7.4 (3.2;11.6)**	−1.1 (−2.7; 0.6)	−1.1 (−2.8; 0.6)	39 (13; 65)**	23 (−6; 52)
Sensitized and exposed to more than one pollen species	18.1 (12.9; 23.3)***	17.9 (12.5; 23.3)***	−2.7 (−4.7; −0.7)**	−2.0 (−4.1; 0.1)	127 (87; 168)***	85 (41; 128)***

*Note*: Coefficients are changes in median values, estimated by multivariable quantile regression.

Abbreviation: CI, Confidence interval.

^a^ Adjusted by sex, age, BMI, smoking status, educational level, case‐control status.

^b^ djusted by sex, age, BMI, smoking status, educational level, case‐control status and sensitization to perennial allergens.

*p < 0.05; **p < 0.01; ***p < 0.001.

18.7% of the studied population reported nasal symptoms but no asthma‐like on pollen exposure. An additional 9.1% reported both nasal and asthma‐like symptoms. FeNO and total IgE levels progressively increased from subjects without symptoms on pollen exposure to those reporting only nasal symptoms on pollen‐related exposure and further to subjects reporting both nasal and asthma‐like symptoms, while FEV1/FVC slightly but significantly decreased. The same pattern emerged when nasal and asthma‐like symptoms were separately considered (Table 5).

**TABLE 5:** Association between pollen‐related symptoms and Fraction of exhaled Nitric Oxide (FeNO), Forced expiratory volume in 1 s (FEV1)/forced vital capacity (FVC) and total IgE levels

Variables		FeNO (ppb) *n* = 856		FEV1/FVC *n* = 1067		Total IgE (kU/L) *n* = 787	
	*n* (%)	Median^a^ (p. 25–p. 75)	p*	Median^a^ (p. 25–p. 75)	p*	Median^a^ (p. 25–p. 75)	p*
Seasonal symptoms							
Wheeze			**<0.001**		0.826		**<0.001**
No	1004 (93.8)	15.8 (10.4–25.0)		82.3 (77.8–86.3)		39.2 (15.7–113.0)	
Yes	66 (6.2)	27.2 (14.7–47.1)		79.9 (75.0–84.5)		155.5 (57.3–283.5)	
Tightness in your chest^b^			0.155		0.425		**0.009**
No	1023 (95.7)	15.8 (10.4–25.6)		82.2 (77.7–86.3)		41.0 (16.1–119.0)	
Yes	46 (4.3)	22.5 (14.4–47.2)		80.3 (75.6–85.7)		133.5 (54.4–318.5)	
Short of breath			0.075		0.735		**<0.001**
No	988 (92.3)	15.7 (10.4–25.1)		82.3 (77.9–86.4)		39.5 (15.4–111.0)	
Yes	82 (7.7)	22.5 (14.6–41.7)		79.4 (74.7–85.1)		158.0 (63.8–358.0)	
Runny or stuffy nose			**0.001**		0.252		**<0.001**
No	781 (73.0)	15.1 (10.0–23.2)		82.5 (78.2–86.6)		32.0 (13.7–97.4)	
Yes	289 (27.0)	20.4 (13.5–41.7)		80.6 (76.1–84.8)		108.5 (46.2–263.5)	
Seasonal nasal and asthma‐like symptoms^b^			**<0.001**		**<0.001**		**<0.001**
No pollen‐related symptom	770 (72.7)	15.1 (10.0–23.2)	Ref.	82.6 (78.2–86.6)	Ref.	32.0 (13.5–96.4)	Ref.
Nasal symptoms only	193 (18.2)	17.8 (12.1–40.2)	**<0.001**	81.0 (76.5–84.8)	**0.004**	94.6 (39.1–239.0)	**<0.001**
Asthma‐like symptoms, with/out nasal symptoms	96 (9.1)	22.7 (14.7–43.0)	**<0.001**	79.6 (75.0–84.8)	**<0.001**	153.0 (60.6–312.0)	**<0.001**

*Note*: Bold signifies seasonal nasal and asthma‐like symptoms: Nasal symptoms only vs. FeNO *p*‐value “<0.001”; vs. FEV1/FVC *p*‐value “0.004”; vs. Total IgE *p*‐value “<0.001”. Seasonal nasal and asthma‐like symptoms: Asthma‐like symptoms, with/out nasal symptoms vs. FeNO; vs. FEV1/FVC; vs. Total IgE, *p*‐value “<0.001”.

^a^ Crude values.

^b^ Information on Tightness in your chest and Seasonal nasal and asthma‐like symptoms was missing in 1 and 11 subjects respectively.

*p‐values were computed by quantile regression, adjusting for case‐control status.

### 3.1 Sensitivity analysis

The association between FeNO levels and exposure/sensitization to one or more pollen species (Supplementary Table 1) and between FeNO levels and symptoms (Supplementary Table 2) were confirmed when FeNO levels were coded as normal (<25 ppb), intermediate (25–50 ppb) and elevated (>50 ppb).

## 4 DISCUSSION

The main results of the present study are:

1. The present study confirms that FeNO levels increase during pollen exposure and in sensitized subjects. The original contribution is the description of the underlying dose‐response relation: FeNO levels are directly related to the number of pollen species around in the previous week and to the number of different pollen species a subject is sensitized to.
2. Moreover, the present study is the first to highlight a strong interaction between sensitization and exposure at population level. With respect to controls, FeNO levels are not significantly higher in subjects sensitized to a pollen species outside the pollen season, or during the pollen season in subjects not sensitized to any pollen species. However, when an individual sensitized to pollen species is exposed to the same species, FeNO levels dramatically increase, especially when several pollen species are involved.
3. FeNO levels increase from controls to subjects with pollen‐related nasal symptom and further to subjects with pollen‐related asthma‐like symptoms.

FeNO levels were directly related to the number of pollen species around in the previous week and to the number of different pollen species a subject is sensitized to. A likely explanation is that in poly‐sensitized subjects IgE with different specificities have an additive effect on the release of mediators from basophils in vitro.^23^ This was confirmed ‘in vivo’ by SPT, showing that the mixture of two different allergens can increase the magnitude of the wheal skin response, in comparison with single allergens.^24^

In agreement with the present study, longitudinal studies, where FeNO was repeatedly measured in asthmatic children allergic to grass or birch pollen, consistently reported a large increase in FeNO levels during the pollen season, which faded away thereafter.^14^, ^25^, ^26^ Also a Swiss study^13^ found that FeNO levels largely increased during natural pollen exposure in pollen‐sensitized subjects. Idrose et al.^27^ investigating an Australian birth cohort at high risk for allergic diseases, recently reported that grass pollen exposure was associated with increased FeNO 1–2 days after exposure, especially in subjects with grass pollen sensitization, asthma or hay fever. Of note, the present study also considered the number of natural pollen species around and the number of allergens a subject was sensitized to.

FENO values are generally reported to be higher in adults with asthma and/or allergic rhinitis (AR) when compared to healthy controls and patients with non‐allergic rhinitis (NAR).^28^ In agreement with our study, FeNO levels were reported to be higher in subjects with both asthma and rhinitis than in those with rhinitis only, and the difference enlarged during the pollen season; this observation was accomplished in a small series of outpatients sensitized to timothy grass, who neither smoke nor were sensitized to house dust mite.^29^ The present study confirmed this observation in a large general population‐based sample; in addition, the present study considered both mono‐ and poly‐sensitized to several allergen species, not only seasonal but also perennial, and took advantage of direct measurement of pollen concentration in the previous week. Of note, FeNO levels were significantly higher in people with symptoms on pollen exposure, particularly in those reporting both nasal and asthma‐like symptoms. This observation further supports the importance of FeNO in the clinical evaluation of allergic subjects, suggesting that FeNO levels are largely increased in people with inflammation involving the whole airways. Accordingly, a study on AR phenotypes found that polyallergic patients have a stronger inflammation and more severe symptoms than mono‐allergic ones.^30^

A Turkish study found a 50% increase in FeNO levels in people with AR with respect to controls, which however was not significant maybe for the limited statistical power.^31^ At variance, a larger study found an increase in FeNO levels in patients with atopic rhinitis with respect to patients with non‐atopic rhinitis or healthy controls.^32^ Accordingly, in the present study FeNO levels were slightly but significantly higher in people with pollen‐related nasal symptoms than without any pollen‐related symptoms. It should be reminded that nasal allergen provocation in patients with AR results in generalized airway inflammation through upregulation of adhesion molecules.^33^

In addition, in the present study the influence of sensitization/exposure to pollen species on FeNO levels was assessed by taking into account potential confounders, such as BMI and smoking habits. The effect of these potential confounders on FeNO levels were in agreement with the current literature. FeNO levels were lower in current smokers than non‐smokers, whereas FeNO levels were higher not only in overweight/obese individuals but also in underweight subjects, with respect to normoweight subjects.^34^, ^35^ As regards demographic characteristics, FeNO levels were higher in men than women, while they did not vary with age in the range considered (20–64 years).

The effect of pollen polysensitization and pollen species‐related exposure was particularly large on FeNO levels and total IgE, while being mild on lung function. It should be reminded that cases and controls were selected from the general population, so that most cases had minor respiratory diseases and controls were subjects who did not refer allergic symptoms during the pollen season but nevertheless could have allergic symptoms when exposed to perennial allergens.

The increase in FeNO levels was not parallelled by a decrease in either FEV1 or Peak Expiratory Flow Rate, in agreement with previous studies.^14^, ^26^ Idrose et al.^27^ found small reductions in FEV1/FVC ratio which were statistically significant but might not be clinically meaningful. This suggests that the allergen‐related airways inflammation took place without significant changes in airways function, and FeNO measurements are far superior to spirometry in order to detect the respiratory effects of pollen exposure in allergic subjects.

## 5 STRENGTHS AND LIMITATIONS

The present study has several strengths. The medical interview, including the questions on symptoms on allergen‐related exposures, was based on the ECRHS questionnaire, a well validated one largely used tool in the epidemiological studies. The studied population was large; subjects were accurately phenotyped by performing SPT to the most common inhaled allergens including pollen species present in the Verona area. Moreover, FeNO levels and lung function were assessed on the same day. Another strength of the study is the objective measurement of the concentration of each pollen considered in the week before clinical evaluation.

However, some limitations should be also acknowledged. First, the design of the present study did not allow to fully assess the temporal relation among pollen species exposure, airway inflammation and lung function decline. Hence, causal‐effect association cannot be inferred. Moreover, the effect of single pollen species on FeNO levels, lung function and allergic symptoms cannot be inferred, as specific inhalation challenge to each single pollen species in poly‐sensitized patients is not feasible.

## 6 CONCLUSIONS

The relation between pollen species exposure/sensitization and FeNO, total IgE levels and FEV1/FVC presented a dose‐response pattern. Co‐exposures and sensitization to more than one pollen species are very common in real life practice, and are likely favoured by climate changes. This should be considered by allergists during natural pollen season in order to evaluate both airway inflammation and pulmonary function. Whenever possible, pollen avoidance strategies should be particularly recommended to poly‐sensitized individuals.

## CONFLICT OF INTEREST

All authors declare no conflict of interest.

## Supporting information

Supplementary Information S1
