# Supplementary material for: Natural pollen exposure increases in a dose‐dependent way Fraction of exhaled Nitric Oxide (FeNO) levels in patients sensitized to one or more pollen species
Source: Clin Transl Allergy. 2022 Feb 6;12(2):e12096. doi: 10.1002/clt2.12096 (PMC8818091; doi:10.1002/clt2.12096)
Supplement: Supplementary file 1 — Supplementary Information S1 [file CLT2-12-e12096-s001.docx]

**Table 1s. Percentage of FeNO levels as a function exposure to pollen species at the time of clinical assessment, and pollen-related sensitization**

| **Variables** |  | **FeNO** | | |  |
| --- | --- | --- | --- | --- | --- |
|  | **n** | **<25 ppb (%)** | **25-50 ppb (%)** | **>50 ppb (%)** | **p** |
| **Exposure to pollen species** |  |  |  |  | **0.003** |
| No pollen around | 239 | 82.4 | 13.0 | 4.6 |  |
| 1 pollen species | 181 | 74.0 | 17.1 | 8.9 |  |
| 2 pollen species | 147 | 70.7 | 17.7 | 11.6 |  |
| ≥3 pollen species | 289 | 66.1 | 22.8 | 11.1 |  |
| **Pollen-related sensitization** |  |  |  |  | **<0.001** |
| No | 471 | 80.9 | 14.4 | 4.7 |  |
| Mono-sensitization | 134 | 73.9 | 18.6 | 7.5 |  |
| Multi-sensitization | 251 | 58.2 | 24.3 | 17.5 |  |

**Table 2s.Percentage of FeNO levels in association with pollen-related symptoms**

| Variables |  | FeNO | | |  |
| --- | --- | --- | --- | --- | --- |
|  | **n** | **<25 ppb (%)** | **25-50 ppb (%)** | **>50 ppb (%)** | **p** |
| Seasonal symptoms |  |  |  |  |  |
| Wheeze |  |  |  |  | **<0.001** |
| No | 804 | 74.9 | 16.9 | 8.2 |  |
| Yes | 52 | 46.2 | 34.6 | 19.2 |  |
| Tightness in your chest^#^ |  |  |  |  | 0.060 |
| No | 818 | 73.7 | 17.9 | 8.4 |  |
| Yes | 37 | 59.5 | 21.6 | 18.9 |  |
| Short of breath |  |  |  |  | **0.006** |
| No | 791 | 74.5 | 17.3 | 8.2 |  |
| Yes | 65 | 56.9 | 26.2 | 16.9 |  |
| Runny or stuffy nose |  |  |  |  | **<0.001** |
| No | 631 | 78.7 | 15.4 | 5.9 |  |
| Yes | 225 | 57.3 | 25.3 | 17.4 |  |
| Seasonal nasal and asthma-like symptoms^#^ |  |  |  |  | **<0.001** |
| No pollen-related symptom | 624 | 78.9 | 15.2 | 5.9 |  |
| Nasal symptoms only | 147 | 58.5 | 23.8 | 17.7 |  |
| Asthma-like symptoms, with/out nasal symptoms | 78 | 55.1 | 28.2 | 16.7 |  |

^#^Information on Tightness in your chest and Seasonal nasal and asthma-like symptoms was missing in one and seven subjects respectively.
